# Characterization of the Mitochondrial Genome of *Cavariella salicicola*: Insight into the Codon Usage Bias and Phylogenetic Implications in Aphidinae

**DOI:** 10.3390/genes16121427

**Published:** 2025-11-29

**Authors:** Tian-Xing Jing, Yan-Jin Zhang, Pei-Xuan Li, Qian Wang, Jin Yang, Hong-Hua Su, Shuai Zhang

**Affiliations:** 1 College of Plant Protection, Yangzhou University, Yangzhou 225009, China; jingtx@yzu.edu.cn (T.-X.J.); m15305162061@163.com (Y.-J.Z.); m17372724320@163.com (P.-X.L.); wq15772161852@163.com (Q.W.); mz120231463@stu.yzu.edu.cn (J.Y.); susugj@126.com (H.-H.S.); 2 Jiangsu Province Engineering Research Center of Green Pesticides, Yangzhou University, Yangzhou 225009, China

**Keywords:** *Cavariella*, mitochondrial genome, codon usage bias, phylogeny

## Abstract

**Background**: *Cavariella salicicola* (Hemiptera: Aphidinae) is a pest on *Salix* spp. and various Umbelliferae (Apiaceae) vegetables. However, the taxonomic status and phylogenetic relationship of the genus *Cavariella* within Aphidinae remain controversial due to the small body size and easily confused external morphology. **Methods**: The complete mitochondrial genome of *C. salicicola* collected from *Oenanthe javanica* was sequenced using the Illumina platform and compared with *C*. *theobaldi.* The codon usage bias of two *Cavariella* aphids was assessed through Enc plot, PR2 plot, and neutrality plot analyses. Furthermore, phylogenetic trees were constructed based on both Maximum Likelihood and Bayesian Inference analysis. **Results**: The *C. salicicola* mitochondrial genome comprises 15,720 bp and represents a typical circular DNA molecule with a high AT content of 83.8%. It contains the standard 37 genes, including 2 ribosomal RNAs (rRNAs), 13 protein-coding genes (PCGs), 22 transfer RNAs (tRNAs), and 2 long non-coding regions (control and repeat regions). Varying degrees of codon usage bias were found across different PCGs, and the bias was predominantly influenced by natural selection rather than mutational pressure. The ratio of nonsynonymous to synonymous substitutions (Ka/Ks) indicated that all PCGs in *C. salicicola*, as well as most other Aphidinae species, are under strong purifying selection. The phylogenetic analysis based on Maximum Likelihood and Bayesian Inference both strongly supported the monophyly of Aphidinae, Macrosiphini, and Aphidini. Crucially, the monophyletic genus *Cavariella* was resolved as a sister group to all other sampled species within the tribe Macrosiphini. **Conclusions**: This study provides new molecular data to support the sister relationship of the genus *Cavariella* to other Macrosiphini aphids. This study will enhance our understanding of phylogenetic relationships within the subfamily Aphidinae.

## 1. Introduction

*Cavariella salicicola*, (Hemiptera: Aphididae), a member of the Macrosiphini tribe, is a polyphagous pest that feeds on numerous *Salix* species and various plants within the Umbelliferae/Apiaceae family, including *Oenanthe* and *Apium* [1,2]. It can transmit several viruses, such as *Celery mosaic virus* and *Konjac mosaic virus*, in a non-persistent manner [3,4]. Despite its economic impact, the biological and genomic information available for this species remains limited. The *Cavariella* genus, which comprises approximately 38 species, is not as speciose as the genus *Aphis*, and nearly half of *Cavariella* species are distributed in Asia. The taxonomic status of *Cavariella* remains contentious, primarily due to uncertain phylogenetic relationships within Macrosiphini and its delineation from related genera like *Pterocomma* [1]. Based on similar morphological characters, host plants, and habitats, Kim et al. proposed that *Cavariella* and *Pterocomma* should be grouped within the subfamily Pterocommatinae [5]. This hypothesis received support from a molecular phylogenetic analysis based on concatenated sequences of partial genes (*cox1*, tRNA + *cox2*, 16S, and *EF-1α*), which revealed that *Cavariella* and *Pterocomma* form a clade that is sister to all other Macrosiphini species [1]. Nevertheless, the phylogenetic position of *Cavariella* within Aphididae is still ambiguous. Therefore, sequencing the complete mitochondrial genome of *C. salicicola* could provide crucial molecular data to resolve these taxonomic uncertainties and facilitate further phylogenetic studies on the genus *Cavariella* and the family Aphididae.

The small body size and easily confused external morphology of aphids have complicated systematic studies within the family Aphididae. Consequently, host alternation, ecological habitats, and molecular marker research have promoted the fields of aphid taxonomy and phylogeny [5,6,7]. Insect mitogenomes have been extensively employed as powerful molecular markers for population genetics analysis and phylogenetic relationship studies [8]. These small-size circular genomes (15–18 kb) are particularly valuable due to their rapid evolutionary rate, limited recombination, and strict maternal inheritance [8,9]. Within the Aphidinae subfamily, the complete mitogenomes of 37 species have been deposited in public databases. Based on these mitochondrial genome data, several taxonomic issues, such as the contentious phylogenetic relationship between the genera *Aphis* and *Toxoptera*, have been resolved [10]. Although several studies have included *Cavariella* in their phylogenetic reconstructions [11,12], the information available for this genus remains limited.

In the present study, we sequenced and characterized the complete mitochondrial genome of *Cavariella salicicola* collected in Yangzhou, China, using high-throughput sequencing. We characterized its fundamental features, including genome structure, nucleotide composition, codon usage bias, evolutionary rate, and phylogenetic relationship. The resulting mitogenome sequence provided new molecular data that will enhance our understanding of phylogenetic relationships within the subfamily Aphidinae.

## 2. Materials and Methods

### 2.1. Aphid Collection and DNA Isolation

An aphid sample of *C. salicicola* was collected from *Oenanthe javanica* from Yangzhou, Jiangsu Province, China, in September 2023. The aphid was identified according to its morphological characteristics and subsequently confirmed by DNA sequence. For details, collected aphids were frozen and pulverized in liquid nitrogen. Then, total genomic DNA was isolated using the DNeasy Blood and Tissue Kit (Qiagen, Hilden, Germany). The integrity and concentration of the extracted DNA were assessed using 1% agarose gel electrophoresis and a NanoDrop 2000 spectrophotometer (Thermo Fisher Scientific, Waltham, MA, USA), respectively. This extracted genomic DNA was used as the template for both PCR amplification (for molecular identification) and subsequent library preparation for mitochondrial genome sequencing.

### 2.2. Sequencing, Assembly, and Annotation

To sequence the mitochondrial genome, a DNA library was constructed and sequenced on an Illumina HiSeq 2500 platform (BIOZERON Co., Ltd., Shanghai, China), generating 2 × 150 bp paired-end reads. This service was provided by Genesky Biotechnologies Inc. (Shanghai, China). The sequencing run produced a total of 802,514 clean reads, representing 119,795,641 bases. The clean reads were assembled (*de novo*) into contigs using metaSPAdes (v3.13.0). The resulting contigs were then mapped to a reference mitochondrial genome from *Aphis gossypii* (NC_024581) to identify mitochondrial sequences. The assembled scaffold was annotated using the MitoMaker pipeline (v1.14) to generate a preliminary mitochondrial genome for *C. salicicola*. Subsequently, we mapped the raw reads back to the assembled genome to obtain the coverage and coverage depth (Supplementary Text S1). This preliminary annotation was subsequently refined using the MITOS web server to accurately identify PCGs, rRNAs, and tRNAs. The boundaries of PCGs and tRNAs were further validated by BLAST (version 2.14.0) searches against homologous genes from closely related aphid species [13]. Additionally, tRNA annotations and their secondary structures were verified using the tRNAscan-SE 2.0 online tool (http://lowelab.ucsc.edu/tRNAscan-SE/, accessed on 5 February 2025) and visualized with the TBI-forna online tool (http://rna.tbi.univie.ac.at/forna/, accessed on 12 July 2025), respectively. The complete annotated mitogenome was visualized as a circular map using the CGView Server (https://proksee.ca/, accessed on 11 August 2025). For comparative analysis, the mitochondrial genome of another *Cavariella* species, *C. theobaldi*, was extracted from whole-genome sequencing data available on NCBI (accession OZ060905.1) and annotated using the same protocol described above.

### 2.3. Nucleotide Diversity, Ka/Ks, and Codon Usage Bias Analysis

The nucleotide base compositions of the individual genes (PCGs and rRNAs), non-coding regions, and whole genome were calculated using MEGA (version X) [14]. The GC skew and AT skew for these regions were determined according to the following formula: GC skew = (G − C)/(G + C), AT skew = (A − T)/(A + T). For PCGs, the relative synonymous codon usage (RSCU) was calculated using Phylosuite v1.2.2 [15,16]. The composition of amino acids of each PCG was calculated using the online tool (https://web.expasy.org/protparam/, accessed on 27 November 2025). Nucleotide diversity (Pi) across the mitogenome was estimated in DnaSP v6.0 with the following sliding-window setting: 200 bp window size; 20 bp step size [17]. The evolution rate of each mitochondrial PCG was evaluated by using the Ka/Ks value (Ka, the nonsynonymous substitution rate; Ks, the synonymous substitution rate). The Ka and Ks values for PCGs in 38 aphid species were calculated in DnaSP v6.0. Subsequently, the values of Ks, Ka, and Ka/Ks were visualized using the ggplot2 package in R v4.3.3 [17].

To characterize the bias of codon usage in PCGs, the CodonW program (version 1.3) and online tools CUSP (https://www.bioinformatics.nl/cgi-bin/emboss/cusp, accessed on 22 July 2025) were used to acquire a series of indices, such as GC3 value (GC content at the third positions of codons), GC3s value (GC content at the third positions of synonymous codons), ENc value (effective number of codons), etc.

The Enc plot was generated to assess the impact of elements on the bias of codon usage. In this plot, the ENc value was set as the y-axis with the x-axis of the GC3s value. The theoretical expected ENc values, indicating neutral evolution, were calculated using the formula: ENc expected value = 2 + GC3s + 29/[GC3s^2^ + (1 − GC3s)^2^] [18].

To evaluate the relative contributions of two major factors (mutation and natural selection) on the bias of codon usage, the Parity Rule 2 (PR2) analysis was conducted. This plot graphs the relationship between A3/(A3 + T3) and G3/(G3 + C3). In this plot, A3, T3, G3, and C3 represent the respective nucleotide frequencies at the 3rd codon position, and the 0.5–0.5 point (center of the plot) indicates no bias [19].

Furthermore, the balance between natural selection and mutation was assessed by constructing the neutrality plot. In this analysis, the GC contents at the 1st and 2nd codon positions (GC12) and at the 3rd codon position (GC3) were calculated using MEGA X [14]. Then, the correlation between GC12 and GC3 values was evaluated and shown as a graph in GraphPad Prism8.

### 2.4. Repeat Elements Analysis

The online software REPuter (https://bibiserv.cebitec.uni-bielefeld.de/reputer/, accessed on 20 July 2025) was used to detect dispersed repeat elements in the mitochondrial genome. Four types of tandem and dispersed repeat elements (forward, reverse, palindromic, and complementary repeats) with more than 30 bp length repeats were searched.

### 2.5. Phylogenetic Analysis

A total of 57 complete aphid mitochondrial genomes, including the *C. salicicola* mitochondrial genome sequenced in this study, 54 mitochondrial genomes from other Aphidinae species, and 2 outgroup sequences from *Therioaphis trifolii* and *Periphyllus diacerivorus*, were downloaded from NCBI (https://www.ncbi.nlm.nih.gov/genbank/, accessed on 22 June 2025). Detailed information for these sequences is provided in Table S1. The sequences and annotations of these mitochondrial genomes were verified and corrected manually because of several errors in the annotations. Individual PCGs and rRNA sequences were fetched from each mitochondrial genome. The 13 PCGs were aligned using MAFFT v7.0 under the Codon Alignment Mode with the L-INS-i strategy. The MAFFT results were then input into MACSE for further optimization with the default parameters. Two rRNAs sequences were aligned separately in MAFFT with the Normal Alignment Mode with the L-INS-I strategy [20]. All aligned PCGs and rRNAs were then processed with Gblocks (version 0.91b) to remove the unreliable gaps [21]. The aligned results of PCGs and rRNAs from all 57 aphids were concatenated into a single supermatrix. This matrix was partitioned into 41 data blocks: 39 blocks corresponding to the three codon positions for each PCGs, and 2 blocks for the two rRNAs (*rrnL* and *rrnS* were treated as separate partitions). The blocked data were then imported into ModelFinder to screen the best-fit partitioning scheme and nucleotide substitution model, employing the greedy search algorithm with linked branch lengths [22]. The best-fit model is shown in Supplementary Text S2.

Both ML (Maximum Likelihood) and BI (Bayesian Inference) analyses were performed based on the final matrix data. For the ML analysis, the best-fit schemes for each 41 partitions recommended by ModelFinder (version 2.2.0) were applied to conduct the ML tree using IQ-tree software (version 2.2.0) with 1000 bootstraps [23]. The BI analysis was performed based on the best-fit schemes as well. Two independent runs of Bayesian Markov Chain Monte Carlo (MCMC) analysis were performed, each with four chains and 10,000,000 generations. Parameters were sampled every 100 generations, and the average deviations of the split frequencies were obtained to achieve a value below 0.01 [24]. The Burn in Fraction was set as 0.25, and the final resulting ML and BI trees were annotated and then visualized using the iTOL online tool (https://itol.embl.de/, accessed on 12 August 2025).

## 3. Results and Discussion

### 3.1. Characterization of the Mitochondrial Genome of C. salicicola

**Figure 1:**
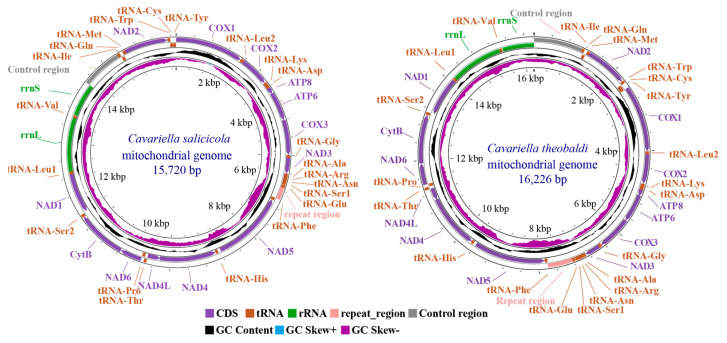
Circular maps of the mitochondrial genomes of *C. salicicola* and *C. theobaldi*. The GenBank ID of *C. salicicola* and *C. theobaldi* is PV837988 and OZ060905.1, respectively. The circular maps were drawn using the CGView Server online tool.

The complete *C. salicicola* mitochondrial genome (GenBank ID: PV837988) was a circular, double-stranded DNA molecule with a total length of 15,720 bp. It was notably smaller than the 16,226 bp mitogenome of *C. theobaldi* (OZ060905.1) (Figure 1). Both lengths fell within the typical size range (14–20 kb) reported for most Aphidinae mitogenomes [12,25,26]. Size variation in aphid mitogenomes is primarily attributed to the control region and repeat region, which have high AT contents. These non-coding regions exhibit length polymorphisms not only among species but also among different geographical populations of the same species [27,28]. In *C. salicicola*, the control region was located between *rrnS* and *tRNA-Ile*, while the repeat region lies between *tRNA-Glu* and *tRNA-Phe*. For other regions, the two *Cavariella* mitogenomes exhibited conserved gene arrangement, which is typical among Aphidinae species.

The *C. salicicola* mitochondrial genome contained 37 genes: 13 protein-coding genes (PCGs), 22 transfer RNAs (tRNAs), and 2 ribosomal RNAs (rRNAs). Four PCGs (*nad1*, *nad4*, *nad4l*, and *nad5*), eight tRNAs (*tRNA-Cys*, *Gln*, *His*, *Leu*, *Tyr*, *Phe*, *Pro*, *His*, *Pro*, and *Val*), and both rRNAs were encoded on the minority (N) strand. The remaining genes were located on the majority (J) strand (Table 1).

The *C. salicicola* mitogenome had 6 gene overlaps and 11 intergenic spacers. The overlap lengths ranged from 1 to 20 bp, and the spacer lengths ranged from 1 to 67 bp (Table 1). Although the number of these features varied among species, the genomic locations of the largest overlap and spacer were conserved within Aphidinae [29,30]. The largest overlaps are usually located between two PCGs, *atp8* and *atp6*, and range from 14 to 20 bp [29,31,32]. Similarly, the location of the largest intergenic spacer is consistently located between *nad5* and tRNA-His.

### 3.2. Nucleotide Composition of the C. salicicola Mitochondrial Genome

Consistent with other aphid mitogenomes, *C. salicicola* exhibited a strong AT bias, with a total AT content of 83.8%. The AT content varied considerably across different regions (Table 2). The highest AT content (>90%) was exhibited at the third codon position of PCGs, whereas the lowest (<80%) was found at the second codon position. This pattern is commonly reported in other aphid species, such as *A. citricidus* and *Hyalopterus amygdali* [10,12]. The overall nucleotide composition was characterized by a positive AT skew (0.083), indicating a slight bias toward “A” over “T”, and a negative GC skew (−0.287), reflecting a moderate bias toward “C” over “G”. This strand asymmetry was also evident in specific regions: PCGs and the control region showed a slight bias toward “A” and “C”; tRNAs were biased toward “A” and “G”; rRNAs were A-skewed and G-skewed; and the repeat region was T-skewed and C-skewed. Such nucleotide asymmetry is not unique to aphids but is a common feature in insect mitogenomes, such as Tortricidae moths [33]. Wei et al. [34] and Francino et al. [35] declared that this bias is likely influenced by strand-specific processes during replication, including the deamination of A and C bases.

### 3.3. Protein Coding Genes, Nucleotide Diversity, and Ka/Ks Analysis

The 13 PCGs in the *C. salicicola* mitochondrial genome had a combined length of 11,004 bp, encoding 3668 codons. The length, start codon, and stop codon for each individual PCG are detailed in Table 1. Consistent with other aphids, the longest and shortest PCGs were *nad5* and *atp8*, respectively. All PCGs initiate with an ATN start codon. Specifically, five genes (*cox1*, *cox2*, *atp6*, *nad3*, and *nad2*) use ATA; four (*atp8*, *nad1*, *nad5*, and *nad6*) use ATT; and the remaining four (*cox3*, *cytb*, *nad4*, and *nad4l*) use ATG. For termination, most PCGs use a complete TAA stop codon. However, *cox1*, *nad2*, *nad4*, and *cytb* terminate with an incomplete single “T” residue. The use of a single “T” as a stop codon is a common feature in insect mitogenomes [10]. Ojala et al. have presumed that the single “T” can be polyadenylated into a normal “TAA” stop codon during the post-transcriptional process [36]. In aphids, the incomplete stop codon “T” usually occurs in *nad4* and *cox1*. In *cox1*, two “A” bases follow the single “T” base and form a complete stop codon in several aphid species, such as *A. citricidus*, *Rhopalosiphum nymphaeae*, and *C. salicicola* in this study [10,28]. However, as this “TAA” sequence overlaps with the adjacent tRNA-Leu gene, it remains uncertain whether “TAA” or the single “T” serves as the actual termination signal for *cox1*.

**Figure 2:**
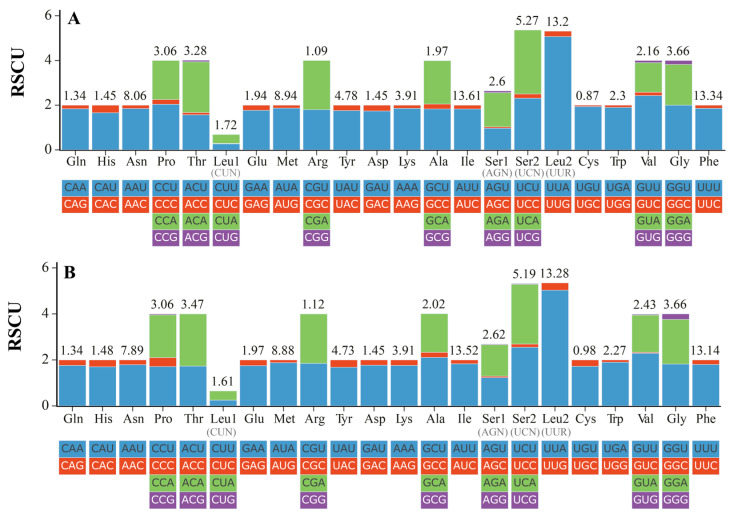
Relative synonymous codon usage (RSCU) in the mitogenomes of two *Cavariella* species. (**A**) The RSCU of the *C. salicicola* mitochondrial genome. (**B**) The RSCU of the *C. theobaldi* mitochondrial genome.

In PCGs, all the 20 amino acids could be found, and the frequency of used amino acids was shown in Figure 2, Table S2. The relative synonymous codon usage (RSCU) of two *Cavariella* aphids presented a similar pattern. Among these coding amino acids, six amino acids (Ile, Phe, Leu2 (UUR), Met, Asn, and Ser2 (UCN)) were the most abundant amino acids with a high frequency value (>5%). In contrast, Cys was the least coding amino acid with a frequency of 0.87%. For the total 55 coding codons used in PCGs, the relative synonymous codon usages (RSCUs) are shown in Figure 2. The UUA(Leu2) had the largest RSCU value (5.07), indicating a strong preference for this codon within the mitogenome [12,37]. The amino acid composition of each PCG is shown in Table S3. Results showed considerable variation in amino acid composition among PCGs. Furthermore, the proportions of energy-intensive amino acids, such as Phe, Trp, and Tyr, were higher in *C. salicicola* than in *C. theobaldi*. This difference implies distinct metabolic loads and resource allocation strategies between these two aphids when under stress. 

**Figure 3:**
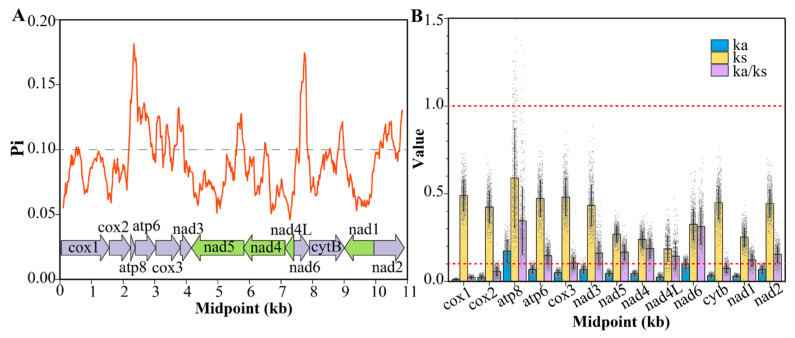
(**A**) Nucleotide diversity (Pi) of 13 PCGs across 38 aphids. (**B**) Ka/Ks values of 13 PCGs across 38 aphids.

The nucleotide diversity (Pi) of PCGs in 38 aphid species was calculated and is shown in Figure 3A. Nucleotide diversity is a key metric for identifying suitable molecular markers in population genetic studies [33,38]. The sliding window analysis revealed considerable variation in Pi among the different PCGs, with average values ranging from 0.058 to 0.148. Four PCGs (*atp8*, *atp6*, *nad6*, and *cox3*) exhibited high nucleotide diversity (Pi > 0.1), indicating their weak stability and their potential in the study of variation evolution [39]. The remaining nine PCGs showed lower variability (Pi < 0.1), indicating stronger purifying selection and greater sequence conservation.

The evolutionary rates of 13 PCGs across 38 aphid species were assessed by calculating nucleotide substitutions, nonsynonymous (Ka), and synonymous sites (Ks) (Figure 3B). The mean Ka/Ks values of all 13 PCGs from 38 aphids were low (<1), indicating a universally purifying selection on all PCGs in aphid species. The lowest three Ka/Ks values were found in *cox1*, *cox2*, and *cytb*, indicating that they were under intense purifying pressure. Consequently, these conserved genes are commonly used as molecular markers for studying deep-level insect evolution [40,41]. In contrast, the *atp8* gene exhibited the highest Ka/Ks ratio among the 13 PCGs, along with the highest frequencies of both nonsynonymous and synonymous substitutions. This elevated evolutionary rate for *atp8* is not unique to aphids but has also been reported in other insects, such as *Piophila casei* and *Hycleus phaleratus* [39,42]. A closer examination of pairwise *atp8* comparisons revealed that while most Ka/Ks ratios were below 1, several exceeded 1. Shi et al. [28] proposed that a single-base deletion, causing a frameshift mutation, could explain such high Ka/Ks values. Although such a deletion was identified in two species, *Schizaphis graminum* (NC_006158.1) and *Hormaphis betulae* (NC_029495.1), the reliability of the *atp8* gene annotations in these species requires further verification.

### 3.4. Codon Usage Bias Analysis

**Figure 4:**
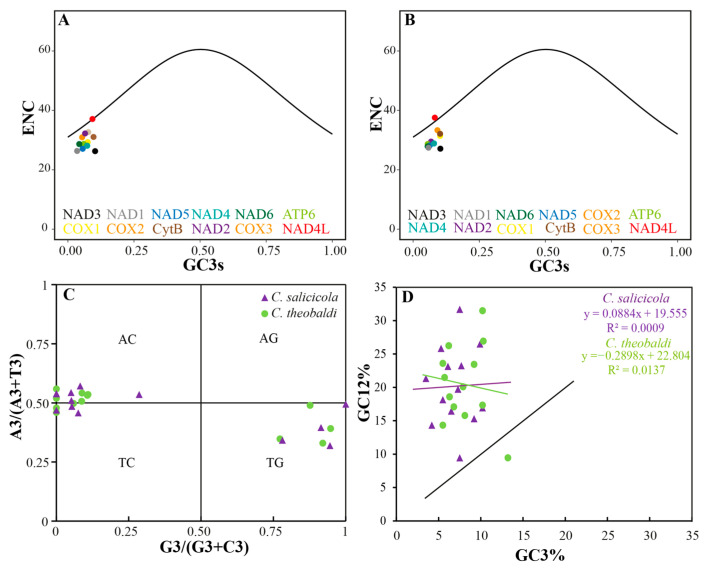
Codon usage bias analysis of aphid mitochondrial genomes. (**A**) Enc plot of PCGs of *C. salicicola* mitochondrial genome. (**B**) Enc plot of PCGs of *C. theobaldi* mitochondrial genome. (**C**) PR2 plot of PCGs from two *Cavariella* mitochondrial genomes. (**D**) Neutrality plot of PCGs from two *Cavariella* mitochondrial genomes.

The ENc (effective number of codons) quantifies the bias extent of codon usage within a gene, with theoretical values ranging from 20 to 61. The minimum ENc value (20) represents the extreme bias: only one synonymous codon is used for each amino acid. In contrast, the maximal ENc value (61) reflects equal usage of all synonymous codons, indicating no bias [18,43]. In *C. salicicola*, ENc values ranged from 26.27 *(nad3*) to 37.09 (*nad4l*), while in *C. theobaldi*, they ranged from 27.10 (*nad3*) to 37.55 (*nad4l*). The GC3s values (GC content at the third synonymous codon positions) ranged from 0.035 *(nad1*) to 0.103 (*nad3*) in *C. salicicola* and from 0.055 (*atp6*) to 0.103 (*nad3*) in *C. theobaldi*. The Enc plot, also known as the ENc-GC3s plot, is a scatter plot with the GC3s value on the abscissa axis and ENc value on the vertical axis. It was used to assess the primary factors shaping codon usage patterns in the two *Cavariella* species. If mutation pressure were the sole influencing factor, all points would lie on or near the standard expected curve [44]. As shown in Figure 4A,B, the points for both *C. salicicola* and *C. theobaldi* exhibited a similar distribution. The *nad4l* gene in both species was located on or near the expected curve, suggesting that its codon usage was primarily influenced by mutational pressure. In contrast, most other PCGs, particularly *nad3*, were situated far below the standard curve. This indicated that natural selection is the dominant force shaping codon usage bias in these genes [18].

The PR2 plot (Parity rule 2 bias plot) is a scatter plot with the y-axis of A3/(A3  +  T3) and the x-axis of G3/(G3  +  C3). The axes were divided into four quadrants and centered on the point of 0.5, 0.5, which represents the state that G = C and A = T. The gene point relative to the central point (0.5, 0.5) indicates the direction and degree of its codon usage bias. Under pure mutational pressure, base composition is expected to be balanced, placing points at the center. Deviation from this point signifies the influence of other factors, such as natural selection, on codon usage [45,46]. As shown in Figure 4C, the PCGs of both *C. salicicola* and *C. theobaldi* were distributed across three quadrants (AC, TC, TG), with no points in the AG quadrant. In *C. salicicola*, six genes (*atp6*, *cox3*, *cytb*, *nad2*, *nad3*, *nad6*) were located in the AC quadrant; three (*atp8*, *cox1*, *cox2*) in the TC quadrant; and four (*nad1*, *nad4*, *nad4l*, *nad5*) in the TG quadrant. Notably, several genes (e.g., *atp8*, *nad2*, *nad3*) in both species were positioned directly on the y-axis, indicating a significant bias specifically between C and G. For *C. theobaldi*, the locations of PCGs in quadrants were generally the same as *C. salicicola*. It is worth noting that several genes were located on the y-axis in both *C. salicicola* and *C. theobaldi*, such as *atp8*, *nad2*, and *nad3*, indicating the significant preference between “C” and “G” bases. The uneven distributions of PCGs and their considerable distance from the central point showed a strong imbalance of A/T and G/C usage. This pattern strongly suggested that codon usage bias in these species was not solely shaped by mutational pressure but was primarily influenced by natural selection [47].

The neutrality plot is drawn based on the GC12 (y-axis) and GC3 (x-axis) values to assess the relationship between these two parameters. According to neutral evolution theory, mutations occur randomly and are assumed to have minimal impact on fitness, affecting all codon positions equally [48,49]. A strong correlation and a regression slope close to 1 would indicate that codons are primarily governed by mutation pressure. Conversely, a weak correlation and a slope deviating from 1 suggest that natural selection exerts a stronger influence on codon usage than mutation pressure [46,50]. Results of the neutrality plot for both *Cavariella* species are presented in Figure 4D. In *C. salicicola*, GC12 ranged from 9.45% (*atp8*) to 26.50% (*cytb*), while GC3 ranged from 3.5% (*nad1*) to 10.2% (*nad3*). Similarly, in *C. theobaldi*, GC12 ranged from 9.45% (*atp8*) to 26.95% (*cytb*) and GC3 ranged from 5.5% (*atp6*) to 13.2% (*atp8*). For both species, the points showed a weak correlation and were distributed far from the diagonal. For details, the regression equation of *C. salicicola* is y = 0.0884x + 19.555, with a low R^2^ of 0.0009; the regression equation of *C. theobaldi* is y = −0.2898x + 22.804, with a low R^2^ of 0.0137. Consistent with the findings from the PR2 plot and Enc plot analysis, the neutrality plot suggested that codon usage patterns in these two aphids deviate significantly from the neutral mutation model. This provided strong evidence that natural selection was the predominant force shaping codon usage bias in mitochondrial PCGs.

### 3.5. Transfer and Ribosomal RNA Genes (tRNA and rRNA)

Consistent with other reported aphids, the *C. salicicola* mitochondrial genome contained 22 tRNA and two rRNA genes. The 16S rRNA (*rrnL*) and 12S rRNA (*rrnS*) were both encoded on the N strand (-strand) with lengths of 1258 bp and 776 bp, respectively. Across Aphidinae, the 16S rRNA varies from 1245 bp (*A. gossypii*, GenBank ID: MW048625.1) to 1260 bp (*Melanaphis donacis*, GenBank ID: OR625201.1); the 12S rRNA varies from 765 bp (*A. citricidus*, GenBank ID: NC_043903.11) to 798 bp (*Melanaphis sacchari*, GenBank ID: MW811104.1).

**Figure 5:**
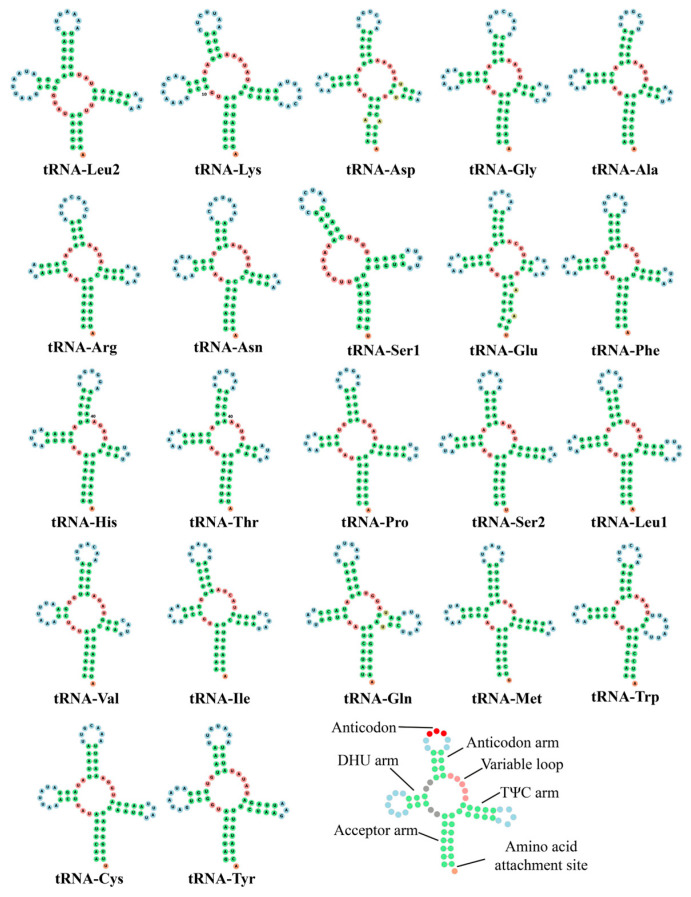
Predicted secondary structures of tRNAs in *C. salicicola* mitochondrial genome.

The locations and arrangements of these 22 tRNAs were conserved in most aphids, such as *A. gossypii* and *R. nymphaeae* [28,51]. The lengths of these tRNAs ranged from 60 bp (*tRNA-Ser1*, GCU) to 73 bp (*tRNA-Lys*). As is common in all metazoan mitochondrial genomes, Ser and Leu amino acids were each decoded by two tRNA genes with different anticodons: *tRNA-Ser1* (GCU), *tRNA-Ser2* (UGA), *tRNA-Leu1* (UAG), and *tRNA-Leu2* (UAA). All tRNAs were predicted to fold into the typical cloverleaf secondary structure model, with the exception of *tRNA-Ser1*, which missed the DHU (dihydrouridine) arm (Figure 5).

### 3.6. Control Region and Repeat Region

**Figure 6:**
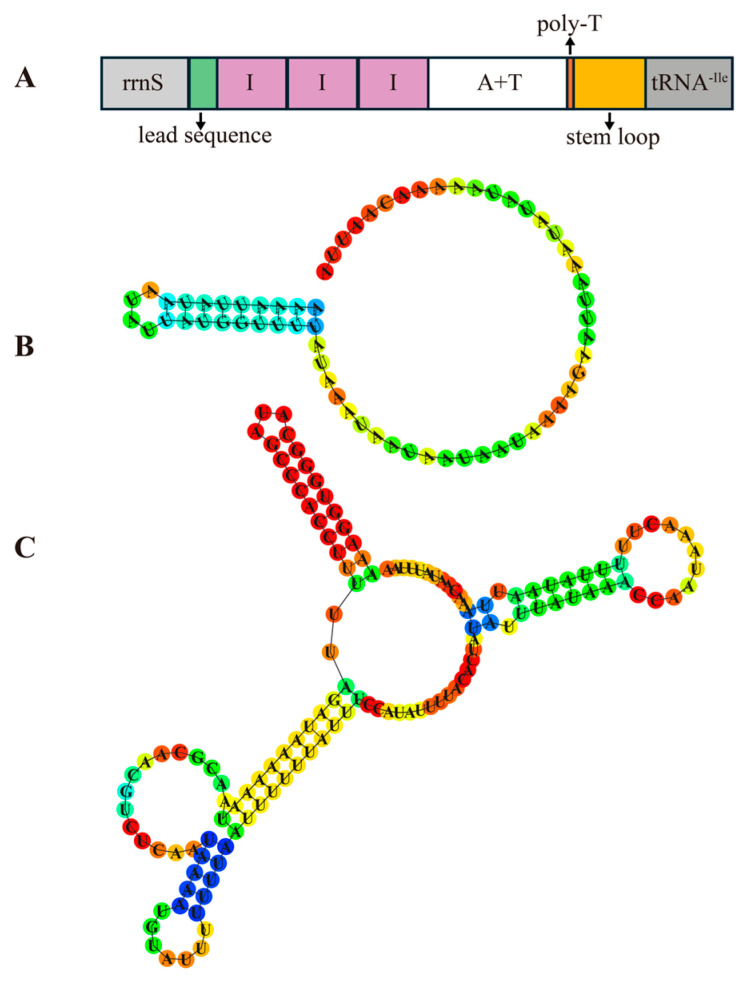
Structure of the control region of the *C. salicicola* mitochondrial genome. (**A**) Organization of control region. (**B**) The predicted secondary structure of the lead sequence in the control region. (**C**) The predicted secondary structure of the stem loop.

A long non-coding RNA with a high AT content region, commonly identified as the control region, has been reported in all known aphid mitochondrial genomes. Typically located between *tRNA-Ile* and *rrnS*, this region ranges from 430 bp (*Sitobion avenae*, NC_024683.1) to 2531 bp (*Myzus persicae*, NC_029727.1). Despite this length variation, the control region is likely conserved in its function. It is involved in mitochondrial DNA replication and promotion of transcription [10,12]. In *C. salicicola*, the control region was 916 bp in length and comprised a 65 bp length lead sequence, three 109 bp tandem repeats, a 375 bp AT-rich region, a 13 bp length poly-T stretch, and a 137 bp stem-loop region (Figure 6). Both lead sequence and stem-loop region could form stable secondary structures. The varieties in the control region size were not only observed across different species but also within the same aphid species from different geographical populations. For instance, the control region of *A. gossypii* from Korea is 797 bp, whereas a shorter 627 bp region was identified in a population from Anyang, China [51,52]. This size variation primarily results from differences in the length and number of tandem repeats. Consistently, a previous study reported a larger 1137 bp control region in a *C. salicicola* population from Gansu, China, which contained five complete 109 bp tandem repeats and a partial anterior repeat unit [2].

Another aphid-specific structural feature in the mitogenome is a long non-coding region between tRNA-Phe and tRNA-Glu, commonly designated the “repeat region” due to the frequent occurrence of tandem repeats [10,28,29]. This region is hypothesized to function as an additional origin of DNA replication in aphid mitochondrial genomes [2,29]. In *C. salicicola*, the 280 bp repeat region contained two copies of a 106 bp tandem unit separated by a 93 bp spacer sequence. In most aphids, the repeat unit is typically 150–300 bp in length and is not interrupted by a spacer. There was a possibility that the *C. salicicola* repeat region only contained one complete 199 bp length repeat unit and one partial repeat unit with only 106 bp. The mitochondrial genome of the Gansu colony (NC_022682.1) has verified our hypothesis. In the repeat region of the Gansu colony, three 199 bp length units were found, and the 106 bp unit and the 93 bp spacer sequence in the Yangzhou colony could be mapped to the 199 bp unit (Figure S1). This finding suggested that *C. salicicola* may have undergone a repeat unit loss or duplication event during population differentiation.

### 3.7. Phylogenetic Analysis

**Figure 7:**
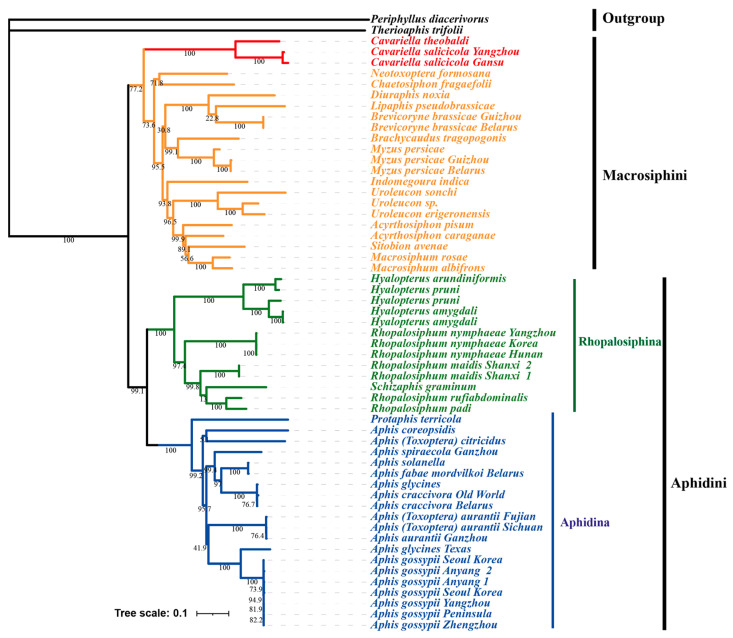
Maximum Likelihood (ML) tree of Aphidinae species based on 13 PCGs + 2 rRNAs. The values near the branches indicate bootstrap probabilities.

**Figure 8:**
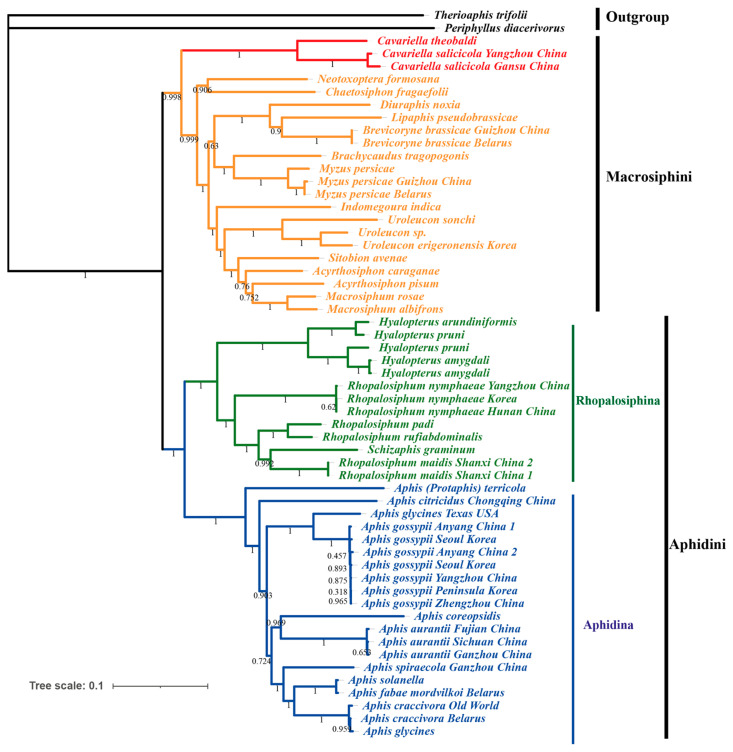
Bayesian Inference (BI) tree of Aphidinae species based on 13 PCGs + 2 rRNAs. The values near the branches indicate posterior probabilities.

In the present study, phylogenetic analysis was conducted using the concatenated dataset (13 PCGs and 2 rRNAs) fetched from 57 published aphid mitochondrial genomes. *Therioaphis trifolii* and *Periphyllus diacerivorus* were set as outgroups to construct both Maximum Likelihood (ML) and Bayesian Inference (BI) trees. The resulting ML and BI trees exhibited congruent topologies with strong support values (Figure 7 and Figure 8). Both analyses confirmed the monophyly of the Aphidinae subfamily and its two constituent tribes, Aphidini and Macrosiphini, which is consistent with previous taxonomic studies. Within Macrosiphini, the ML and BI topological structures are identical with high support values. *Cavariella* is monophyletic and formed as a sister group to all other Macrosiphini species. Choi et al. [1] has demonstrated that several genera traditionally classified within Macrosiphini, such as *Cavariella* and *Liosomaphis*, were closer to Pterocommatini and can form a *Pterocomma* group. Furthermore, several studies have proposed that the clade containing *Pterocomma* and *Cavariella* was the sister to the monophyletic clade of Macrosiphini plus Aphidini [5,53]. In summary, our results support the point that *Cavariella* shares a distant relationship with other Macrosiphini species.

Within the Aphidini tribe, Aphidina and Rhopalosiphina were two clearly monophyletic groups. The *Hyalopterus* genus was monophyletic and positioned as a sister to the monophyletic clade of *Rhopalosiphum* + *Schizaphis*, as seen in a previous study [12]. Within Aphidina, all *Aphis* species, including *Aphis* (*Toxoptera*) *citricidus* and *Aphis* (*Toxoptera*) *aurantia*, formed a monophyletic clade. This result supported the idea that *Toxoptera* should be corrected as *Aphis* [7,10,54]. Conversely, the aphid from the *Protaphis* genus, which was classified as a subgenus of *Aphis* before [55,56], was placed outside of the core *Aphis* clade. The *Protaphis* has been elevated to genus level before, and several *Protaphis* species were transferred into *Aphis* to form a subgenus, *Pseudoprotaphis* [56,57,58]. However, the relationship between *Protaphis* and *Aphis* is still unclear because of the lack of complete mitochondrial genome data of *Protaphis.* Thus, further studies on mitochondrial genomes of aphids are essential to clarify the relationship within Aphidinae.

## 4. Conclusions

The complete mitochondrial genome of *Cavariella salicicola* was obtained via high-throughput sequencing. The circular genome is 15,720 bp in length and exhibits conserved gene organization and nucleotide composition as other Aphidinae species. Various degrees of codon usage bias across different PCGs were observed, and results indicated that natural selection is the primary factor influencing this bias. Phylogenetic analyses based on 57 complete mitogenomes supported the monophyly of the Aphidinae subfamily and its two constituent tribes, Aphidini and Macrosiphini. Furthermore, both Maximum Likelihood and Bayesian Inference trees supported that the monophyletic *Cavariella* may be a sister group to all other Macrosiphini species.

## Figures and Tables

**Table 1:** Annotation and gene organization of the *C. salicicola* mitochondrial genome.

Gene/Region	Strand	Position	Size	Start Codon	Stop Codon	Anticodon	Intergenic
*tRNA-Ile*	J	1–64	64			GAU	0
*tRNA-Gln*	N	62–127	66			UUG	−3
*tRNA-Met*	J	133–198	66			CAU	5
*nad2*	J	199–1174	976	ATA	T		0
*tRNA-Trp*	J	1175–1236	62			UCA	0
*tRNA-Cys*	N	1229–1296	68			GCA	−8
*tRNA-Tyr*	N	1300–1364	65			GUA	3
*cox1*	J	1366–2896	1531	ATA	T		1
*tRNA-Leu2*	J	2897–2964	68			UAA	0
*cox2*	J	2965–3639	675	ATT	TAA		0
*tRNA-Lys*	J	3642–3714	73			CUU	2
*tRNA-Asp*	J	3715–3778	64			GUC	0
*atp8*	J	3779–3937	159	ATT	TAA		0
*atp6*	J	3918–4571	654	ATT	TAA		−20
*cox3*	J	4571–5355	785	ATG	TA		0
*tRNA-Gly*	J	5356–5419	64			UCC	0
*nad3*	J	5420–5773	354	ATA	TAA		0
*tRNA-Ala*	J	5774–5836	63			UGC	0
*tRNA-Arg*	J	5836–5899	64			UCG	−1
*tRNA-Asn*	J	5900–5963	64			GUU	0
*tRNA-Ser1*	J	5963–6022	60			GCU	−1
*tRNA-Glu*	J	6030–6095	66			UUC	7
Repeat region	J	6096–6369	274				0
*tRNA-Phe*	N	6370–6433	64			GAA	0
*nad5*	N	6434–8155	1722	ATT	TAA		0
*tRNA-His*	N	8223–8283	61			GUG	67
*nad4*	N	8284–9607	1324	ATG	T		0
*nad4l*	N	9601–9891	291	ATA	TAA		−7
*tRNA-Thr*	J	9893–9954	62			UGU	1
*tRNA-Pro*	N	9957–10,022	66			UGG	2
*nad6*	J	10,024–10,515	492	ATT	TAA		1
*cytb*	J	10,519–11,632	1114	ATG	T		3
*tRNA-Ser2*	J	11,633–11,697	65			UGA	0
*nad1*	N	11,708–12,643	936	ATT	TAA		10
*tRNA-Leu1*	N	12,644–12,708	65			UAG	0
*rrnL*	N	12,709–13,966	1258				0
*tRNA-Val*	N	13,967–14,028	62			UAC	0
*rrnS*	N	14,029–14,804	776				0
Control region	J	14,805–15,720	916				0

J, majority strand; N, minority strand.

**Table 2:** Nucleotide composition of the *C. salicicola* mitochondrial genome.

Regions	Size (bp)	AT Content (%)	GC Content (%)	AT Skew	GC Skew
Full length	15,720	83.8	16.2	0.083	−0.287
PCGs	11,004	83.1	16.9	−0.152	−0.055
PCGs–1st codon	3668	80.2	19.8	0.005	0.127
PCGs–2nd codon	3668	75.9	24	−0.391	−0.127
PCGs–3rd codon	3668	93.3	6.8	−0.093	−0.331
PCGs–J strand	6729	81.8	18.2	−0.051	−0.252
PCGs–J strand–1st codon	2243	78.9	21.2	0.124	−0.008
PCGs–J strand–2nd codon	2243	73.7	26.3	−0.346	−0.27
PCGs–J strand–3rd codon	2243	92.7	7.3	0.034	−0.89
PCGs–N strand	4275	85.2	14.7	−0.305	0.329
PCGs–N strand–1st codon	1425	82.4	17.7	−0.175	0.381
PCGs–N strand–2nd codon	1425	79.4	20.5	−0.458	0.16
PCGs–N strand–3rd codon	1425	94.1	5.9	−0.29	0.762
tRNAs	1422	84.8	15.1	0.031	0.181
*rrnL*	1258	84.9	15.1	−0.122	0.4
*rrnS*	765	83.5	16.5	−0.039	0.302
control region	916	85.5	14.4	−0.013	−0.348
repeat region	274	91.6	8.4	0.116	−0.391

## Data Availability

The data that support the findings of this study are openly available in GenBank with accession number of PV837988.

## Supplementary Materials

The following supporting information can be downloaded at https://www.mdpi.com/article/10.3390/genes16121427/s1. Figure S1: Repeat region in two mitochondrial genomes from different populations of *C. salicicola*; Table S1: Detail information of mitochondrial genomes used in this study; Table S2: Relative synonymous codon usage of *Cavariella salicicola*; Table S3: Amino acid composition of PCGs in *C. salicicola* and *C. theobald*; Supplementary Text S1: sequence coverage depth; Supplementary Text S2: Best-fit model used in phylogenetic analysis.
