# Supplementary material for: Characterization of the Mitochondrial Genome of Cavariella salicicola: Insight into the Codon Usage Bias and Phylogenetic Implications in Aphidinae
Source: Genes (Basel). 2025 Nov 29;16(12):1427. doi: 10.3390/genes16121427 (PMC12732342; doi:10.3390/genes16121427)
Supplement: Supplementary file 1 [file genes-16-01427-s001.zip › Supplementary Materials/Supplementary Materials.pdf]

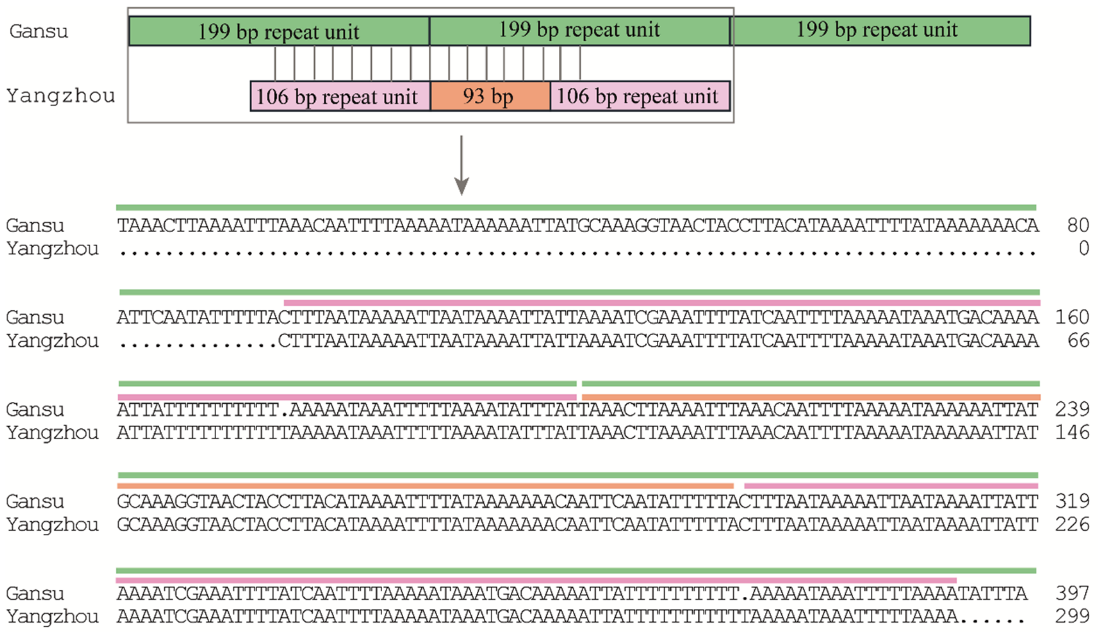

Figure S1. Repeat region in two mitochondrial genomes from different populations of *C. salicicola*

Table S1. Detail information of mitochondrial genomes used in this study

GenBank ID	Organism
PQ096067.1	*Uroleucon sp.*
MT533446.1	*Uroleucon sonchi*
MZ695840.1	*Uroleucon erigeronensis*
NC_024683.1	*Sitobion avenae*
NC_006158.1	*Schizaphis graminum*
NC_062327.1	*Rhopalosiphum rufiabdominalis*
KT447631.1	*Rhopalosiphum padi*
NC_046740.1	*Rhopalosiphum nymphaeae*
OM214586.1	*Rhopalosiphum nymphaeae*
MZ420705.1	*Rhopalosiphum nymphaeae*
OR148359.3	*Rhopalosiphum maidis*
NC_081990.1	*Rhopalosiphum maidis*
OZ060891.1	*Protaphis terricola*
NC_057970.1	*Neotoxoptera formosana*
NC_029727.1	*Myzus persicae*
MN232006.1	*Myzus persicae*
KU877171.1	*Myzus persicae*
MW811104.1	*Melanaphis sacchari*
OR625201.1	*Melanaphis donacis*
NC_064372.1	*Macrosiphum rosae*
NC_063971.1	*Macrosiphum albifrons*

NC_072150.1	*Lipaphis pseudobrassicae*
NC_045897.1	*Indomegoura indica*
OK641614.1	*Hyalopterus pruni*
NC_050904.1	*Hyalopterus pruni*
OK274075.1	*Hyalopterus arundiniformis*
OZ060888.1	*Hyalopterus amygdali*
OK641613.1	*Hyalopterus amygdali*
NC_022727.1	*Diuraphis noxia*
DQ021446.1	*Daktulosphaira vitifoliae*
LC590896.1	*Chaetosiphon fragaefolii*
OZ060905.1	*Cavariella theobaldi*
NC_022682.1	*Cavariella salicicola*
PV837988	*Cavariella salicicola*
NC_056270.1	*Brevicoryne brassicae*
MW267824.1	*Brevicoryne brassicae*
OZ060884.1	*Brachycaudus tragopogonis*
NC_053819.1	*Aphis spiraecola*
NC_068764.1	*Aphis solanella*
OR449278.1	*Aphis gossypii*
NC_024581.1	*Aphis gossypii*
MK994521.1	*Aphis gossypii*
MW048625.1	*Aphis gossypii*
MW013764.1	*Aphis gossypii*
MT430940.1	*Aphis gossypii*
MN102349.1	*Aphis gossypii*
NC_045236.1	*Aphis glycines*
KT889380.1	*Aphis glycines*
NC_039988.1	*Aphis fabae mordvilkoi*
NC_031387.1	*Aphis craccivora*
MT095075.1	*Aphis craccivora*
NC_068763.1	*Aphis coreopsidis*
NC_043903.1	*Aphis citricidus*
NC_052865.1	*Aphis aurantii*
OM350401.1	*Aphis aurantii*
MN397939.1	*Aphis aurantii*
KP722589.1	*Adelges laricis*
NC_011594.1	*Acyrthosiphon pisum*
NC_064371.1	*Acyrthosiphon caraganae*

Table S2. Relative synonymous codon usage of *Cavariella salicicola*

AA	Codon	Count	RSCU	aaRatio

Ala	GCU	33	1.83	
Ala	GCC	4	0.22	
Ala	GCA	35	1.94	
Ala	GCG	0	0	1.97
Cys	UGU	31	1.94	
Cys	UGC	1	0.06	0.87
Asp	GAU	46	1.74	
Asp	GAC	7	0.26	1.45
Glu	GAA	63	1.77	
Glu	GAG	8	0.23	1.94
Phe	UUU	453	1.86	
Phe	UUC	35	0.14	13.34
Gly	GGU	67	2	
Gly	GGC	0	0	
Gly	GGA	61	1.82	
Gly	GGG	6	0.18	3.66
His	CAU	44	1.66	
His	CAC	9	0.34	1.45
Ile	AUU	455	1.83	
Ile	AUC	43	0.17	13.61
Lys	AAA	133	1.86	
Lys	AAG	10	0.14	3.91
Leu2	UUA	461	5.07	
Leu2	UUG	22	0.24	13.2
Leu1	CUU	25	0.27	
Leu1	CUC	2	0.02	
Leu1	CUA	36	0.4	
Leu1	CUG	0	0	1.72
Met	AUA	306	1.87	
Met	AUG	21	0.13	8.94
Asn	AAU	273	1.85	
Asn	AAC	22	0.15	8.06
Pro	CCU	57	2.04	
Pro	CCC	6	0.21	
Pro	CCA	49	1.75	
Pro	CCG	0	0	3.06
Gln	CAA	45	1.84	
Gln	CAG	4	0.16	1.34
Arg	CGU	18	1.8	
Arg	CGC	0	0	
Arg	CGA	22	2.2	
Arg	CGG	0	0	1.09
Ser2	UCU	83	2.31	
Ser2	UCC	7	0.19	

Ser2	UCA	103	2.86	
Ser2	UCG	0	0	5.27
Ser1	AGU	35	0.97	
Ser1	AGC	2	0.06	
Ser1	AGA	55	1.53	
Ser1	AGG	3	0.08	2.6
Thr	ACU	47	1.57	
Thr	ACC	3	0.1	
Thr	ACA	68	2.27	
Thr	ACG	2	0.07	3.28
Val	GUU	48	2.43	
Val	GUC	3	0.15	
Val	GUA	26	1.32	
Val	GUG	2	0.1	2.16
Trp	UGA	80	1.9	
Trp	UGG	4	0.1	2.3
Tyr	UAU	154	1.76	
Tyr	UAC	21	0.24	4.78
